# Upper limb disability in Norwegian workers with hand-arm vibration syndrome

**DOI:** 10.1186/1745-6673-9-5

**Published:** 2014-02-11

**Authors:** Kristin Buhaug, Bente Elisabeth Moen, Ågot Irgens

**Affiliations:** 1 Department of Occupational Medicine, Haukeland University Hospital, Bergen, Norway; 2 Department of Global Public Health and Primary Care, University of Bergen, Bergen, Norway

**Keywords:** HAVS, Hand-arm vibration, DASH questionnaire, Tendinitis, Stockholm Workshop scales

## Abstract

**Background:**

Hand-arm vibration syndrome (HAVS) is a well-known disease among workers using hand-held vibrating tools. These patients experience major symptoms from their upper limbs. However, there are few studies on disability in this patient group. In this study we wanted to describe the disability of HAVS patients.

**Methods:**

All HAVS patients diagnosed at Haukeland University Hospital in Bergen, Norway in a five-year period were invited. The disabilities of the arm, shoulder and hand (DASH) questionnaire was sent by mail. Clinical data were extracted from their hospital journals. Descriptive statistics and regression analyses were performed.

**Results:**

Thirty-eight patients were recruited. Mean DASH score was 41.2, while the mean of a normal population is 10. Ability to perform tasks related to work and everyday life was affected in these patients. We found a significant association between the DASH score, hand grip strength and tendinitis, also after adjustment for age and smoking in pack-years.

**Conclusion:**

HAVS patients demonstrate a high level of upper limb disability as assessed by the DASH score. Ability to perform tasks related to work and everyday life was affected. We found a significant association between the DASH score, hand grip strength and tendinitis. This should be focused upon in future research.

## Background

Hand-arm vibration syndrome (HAVS) denotes health effects associated with occupational exposure to hand-held vibrating tools [1]. There are different opinions about the syndrome, but patients with HAVS are most commonly described as having neurological, vascular and musculoskeletal symptoms and signs such as disturbed sensation (numbness, tingling), cold intolerance, episodic finger blanching, pain and weakness in hands and arms [2,3]. The condition is heterogeneous with a continuum of symptoms and signs from workers displaying acute effects of vibration to patients with established HAVS.

In Norway, HAVS has been recognized as a work-related disease for decades, but no clinical research has been published since 1972 [4]. As a result, teaching of medical students and occupational health personnel has been lacking. Consequently, preventive measures on this issue are not well known at Norwegian work places. According to a national survey conducted by Statistics Norway in 2009, five per cent of the working population is exposed daily to hand-arm vibration at least 25% of the day [5]. Workers most often exposed include mechanics, welders, platers, carpenters, and road and construction workers. This is in agreement with studies from other countries [6]. Taking into account the Norwegian working force this corresponds to more than 100 000 Norwegian workers being at risk of developing HAVS.

Workers with HAVS examined at the Department of Occupational Health in Health Region West (1 million inhabitants) often have a history of many years of symptoms prior to seeking a doctor. Little attention has been given to the condition by their company doctor or general practitioner, both regarding preventive measures and treatment options. As there is little treatment to offer these patients apart from symptom relieving measures [7,8], there is reason to intensify the focus on early recognition of the condition.

Most studies on HAVS have focused on the vascular and neurological symptoms and how to assess these. Correspondingly, the Stockholm Workshop scales (SWS) have been given much attention [9-11]. However, many patients describe symptoms not included in these scales; symptoms often classified as “musculoskeletal”. Several studies describe carpal tunnel syndrome (CTS) in connection with HAVS [12,13]. Other musculoskeletal conditions discussed in relation to HAVS include tendinitis in hand, elbow and shoulder [14].

The musculoskeletal symptoms might not be well recognized if the patients are assessed only by the SWS. This is recognized by several authors as a number of studies published over the last decade describe symptoms and clinical outcome related to disability [15-19]. It is apparent that many HAVS patients experience considerable disability both at work and in their spare time [20,21]. One instrument used in these studies is the self-administered: The disabilities of the arm, shoulder and hand questionnaire (DASH). This is designed to measure upper extremity disability and symptoms [22]. It is translated to a number of languages; among them the Norwegian version which is based on the Swedish questionnaire [23]. The latter is evaluated and found to be a reliable and valid instrument in upper-extremity musculoskeletal disorders [24].

A total disability score (the DASH score), based on the answers to all 30 items in the questionnaire, is used in a large number of studies on diverse populations, among these, HAVS patients. To our knowledge, no study describes the result of individual items in the questionnaire.

The aim of this study was to describe disability in the upper limbs of HAVS patients, by using the DASH questionnaire. In addition to calculating the DASH -score, we wanted to describe the score for each answer in the questionnaire. This could give us a more thorough impression of the extent of the disability experienced by this patient group. Secondly, we wanted to study the relationship between DASH score and years of hand-arm vibration exposure, the Stockholm Workshop scales (SWS), objective signs of hand-arm dysfunction, tendinitis and carpal tunnel syndrome.

## Methods

This was an observational study. All patients diagnosed with HAVS at Haukeland University Hospital in Norway in the five-year period from 2003–2008, a total number of 53 persons, were contacted by mail in July 2008 by an occupational physician at the clinic. They were asked permission to use their medical records for research purposes, and they were asked to complete the DASH questionnaire. The diagnosis of HAVS was based on exposure history and presentation of typical vascular and/or neurological findings. Examination of the patients at the Department of Occupational Medicine included an interview and clinical examination, both performed by a team of three occupational physicians; one being a senior supervising the two others.

All available information regarding HAVS was extracted from the medical records. These were based on clinical assessment of the patients in the period from December 2003 to June 2008. Clinical assessment included objective measures of hand-grip strength with Hand dynamometer, and dexterity with Grooved pegboard. The DASH questionnaire was sent to the patients in July 2008.A written consent and the questionnaire were returned to the physician in a pre-paid envelope from the participants. One reminder was sent.

The DASH questionnaire contains 30 items measuring disability in the week prior to completing the questionnaire. Each item has five possible response options, graded on a scale from 1–5. In order to estimate a total score, at least 27 of the 30 questions had to be answered [22]. The total score; the DASH score, was calculated according to the original documentation and missing answers handled as recommended. Higher scores indicated higher disability.

The following data were extracted from the medical record for each patient: Age, gender, number of years at work, number of years exposed to hand-arm vibrations, occupation, current employment status, smoking history, carpal tunnel syndrome and chronic tendinitis in arms/hands. The medical record for each patient contained information regarding smoking habits, i.e. number of years smoking and daily tobacco consumption. This enabled us to calculate the total burden of tobacco exposure in number of pack-years both for current smokers and former smokers. Carpal tunnel syndrome was defined as having a neurophysiologically confirmed diagnosis, with or without surgical treatment.

Tendinitis was defined as symptoms and signs of tendinitis (shoulder, elbow or hand) at clinical examination; distinctive pain on palpation of tendon and painful isometric movement of the muscle in question, and with at least three months history of the condition prior to examination.

Each patient was categorized according to the SWS (Table 1); with both vascular and sensorineural components [9,10]. This estimate was done by comparing subjective symptoms with outcome of the clinical examination, and both hands were scored individually. The results for the worse hand were used in the analysis.

**Table 1:** The Stockholm workshop scale

**Vascular assessment**		**Sensorineural assessment**	
**Stage**	**Description**	**Stage**	**Symptoms**
0	No attacks	0SN	Exposed to vibration but no symptoms
1 V	Occasional attacks affecting only the tips of one or more fingers	1SN	Intermittent tingling with or without tingling
2 V	Occasional attacks affecting distal and middle (rarely also proximal) phalanges of one or more fingers	2SN	Intermittent or persistent numbness reducing sensory perception
3 V	Frequent attacks affecting all phalanges of most fingers	3SN	Intermittent or persistent numbness reducing tactile discrimination and/or manipulative dexterity
4 V	As in stage 3, with trophic skin changes in the finger tips		

Each hand is staged separately.

Clinical examination was performed by an occupational physician, and included measurement of hand-grip strength (Lafayette Hand Dynamometer 78010) and manual dexterity (Grooved Pegboard Test). Reduced hand-grip strength and manual dexterity were defined as t-score 40 or below for his/her age group.

The study was approved by The Regional Committee for Medical and Health Research Ethics Western Norway.

The data were presented using descriptive statistics. Associations between DASH score (dependent variable) and the independent variables years of vibration exposure (≤ 20 years, > 20 years) employment (yes/no) carpal tunnel syndrome (yes/no), tendinitis (yes/no), hand-grip strength (normal/ reduced) and manual dexterity (normal/reduced) were tested by a univariate linear regression analysis. The analyses were performed while adjusting for age and smoking (pack-years). Due to the small number in our study population, we chose to analyse the complete population. Separate analyses where women were excluded, did not alter the results. SPSS version 18.0 was used for the analyses and the significance level was set below 0.05.

## Results

Thirty-eight patients, 31 males and seven females, agreed to participate in the study, a response rate of 72% (Table 2). Of these, 14 patients were assessed in the first two years (Dec 2003- Dec 2005) and 24 patients were assessed in the last 3 year period (March 2006- June 2008). The average age was 49.9 years and they had been exposed to hand-arm vibrating tools for an average duration of 22.4 years. The women were slightly younger and had five years shorter exposure history than the men, but the differences were not significant. Thirty of 38 patients (79%) were smokers at the time of the examination or had been regular smokers in the past.

**Table 2:** **Descriptive characteristics of study participants** (**n** = **38**)

**Variable**	**Men (n = 31)**	**Women (n = 7)**
	**Mean (SD)**	**Mean (SD)**
Age	50 (10)	48 (7)
Vibration exposure in years	23.0 (8.6)	18.0 (5.8)
Smoking (pack-years)	13.3 (11.3)	9.6 (8.8)
	n (%):	n (%):
Current smokers	15 (48.4)	3 (42.9)
Mechanical Industy	14 (45)	6 (86)
Construction Industry	13 (42)	0
Car repair shops	4 (13)	1 (14)
Out of work	17 (55)	2 (29)
Carpal tunnel syndrome	5 (16)	1 (14)
Tendinitis	16 (52)	6 (86)
HAVS vascular		
0	6 (19)	4 (57)
1	0	1 (14)
2	10 (32)	2 (29)
3	15 (48)	0
4	0	0
HAVS neurological		
0	3 (10)	1 (14)
1	2 (6)	0
2	3 (10)	2 (29)
3	23 (74)	4 (57)
Reduced hand grip strength	10 (32)^1^	2 (29)
Reduced manual dexterity	14 (45)^2^	3 (43)^3^

^1^3 missing. ^2^4 missing. ^3^1missing.

The patients were employed in three different industries, where the mechanical industry was the most prevalent (53%). For the women, 6 of 7 were employed in the mechanical industry. Fifty per cent of the patients were out of work due to health problems. Especially for the construction workers, there was a strong tendency to falling out of work, as 62% (8 of 13) no longer worked when the assessment was completed. For the other two industries the figures were 40% (8 of 20) for the mechanical industry and 60% for car repair shops (3 of 5).

The percent of workers in the different Stockholm workshop stages were for the neurological staging (0–3 SN) 11, 5, 13 and 71%, respectively, and for the vascular staging (0–3 V) 26, 2, 32 and 40%, respectively, with none classified as stage 4 V. Thirteen of the patients (34%) were categorized as stage 3 for both scales; 3 V, 3SN (Table 3). According to the staging system this corresponds to severe impairment.

**Table 3:** **Stockholm workshop scale** (**SWS**) - **Vascular and sensorineural staging for the HAVS patients**

		**SWS** – **Sensorineural staging**				**Total (n)**
		**0**	**1**	**2**	**3**	**0**
SWS – Vascular staging	0	0	1	3	6	10
	1	0	0	1	0	1
	2	4	0	0	8	12
	3	0	1	1	13	15
Total (n)		4	2	5	27	38

The numbers corresponds to SWS -staging for each patient.

**Figure 1:**
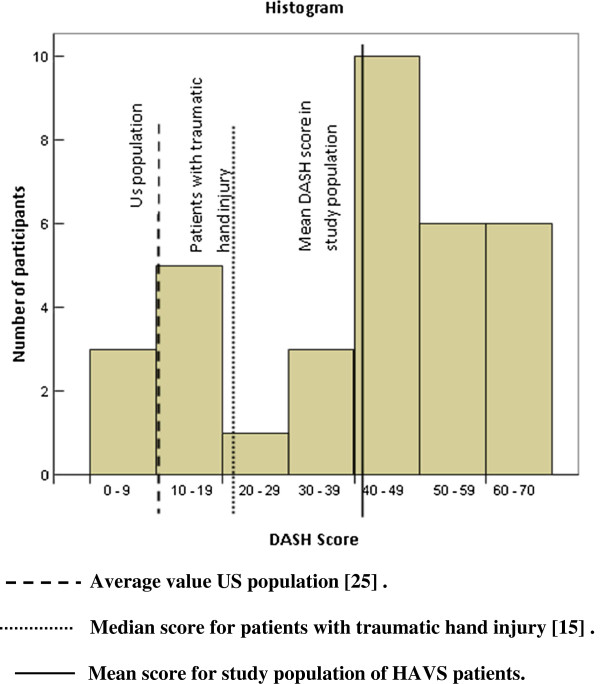
**DASH score in HAVS patients (n = 34).** Mean score is 41.21, median score 45.00. Average value US population [25]. Median score for patients with traumatic hand injury [15] Mean score for study population of HAVS patients.

The DASH questionnaire was received from all 38 patients, but due to missing answers, the score was calculated for only 34. The average score in the study group was 41.2 ± 19.7 (Figure 1). Although not significant, the women in the study had a higher score (51.8) than the men (38.9). The answers to the individual questions indicate that several tasks related to everyday life were affected. More than 50% of the patients had severe difficulty or were unable to perform tasks demanding a certain muscular strength, for instance heavy household chores and recreational activities demanding some force or free movement of the arm. Only 6% of the patients described no limitation in work or regular daily activities. The hand-arm problems also affected the answers to a question reflecting the patients’ self-esteem, as 63% agreed (or strongly agreed) with the statement measuring this quality.

In a linear regression analysis of all the patients (Table 4), the individual variables tendinitis and hand grip strength were significantly related to the DASH score. This was also the case when adjusting for age and smoking (pack-years). The DASH score was not related to vibration exposure, the Stockholm scales, carpal tunnel syndrome or manual dexterity. This was found when analysing both men and women together. Analysing only men gave similar results.

**Table 4:** **Comparisons of DASH score between categories of several covariates**, **and associations between DASH score and continuous covariates**

		**Crude DASH score**	**P-value**	Adjusted DASH score**	**P-value**
**Categorical**		**Mean (SE)**		**Mean (SE)**	
**Vibration exposure**	≤ 20 ys	43.2 (5.0)	Reference	44.3 (5.3)	Reference
	> 20 ys	39.4 (4.7)	0.588	39.4 (5.5)	0.541
**Employment**	Yes	35.0 (4.9)	Reference	34.8 (4.9)	Reference
	No	45.8 (4.6)	0.117	48.6 (5.3)	0.073
**Carpal tunnel syndrome**	No	41.8 (3.8)	Reference	42.8 (4.1)	Reference
	Yes	38.3 (8.1)	0.700	38.5 (8.4)	0.654
**Tendinitis**	No	30.6 (4.5)	Reference	32.0 (4.9)	Reference
	Yes	49.6 (4.0)	0.004	50.1 (4.4)	0.011
**Hand grip strength**	Normal	36.3 (3.9)	Reference	37.6 (4.1)	Reference
	Reduced	52.5 (5.3)	0.020	54.3 (6.0)	0.031
**Manual dexterity**	Normal	37.6 (5.2)	Reference	37.6 (5.3)	Reference
	Reduced	45.9 (5.0)	0.264	48.9 (5.7)	0.159
**SWS vascular**	Stage 0 - 2	38.2 (4.2)	Reference	39.3 (4.4)	Reference
	Stage 3	46.8 (5.6)	0.231	47.4 (6.5)	0.313
**SWS sensorineural**	Stage 0 - 2	37.4 (6.0)	Reference	40.1 (6.5)	Reference
	Stage 3	43.0 (4.1)	0.440	42.8 (4.5)	0.737

*Dependent variable: DASH score. **Adjusted for age and smoking (pack-years), 4 missing.

DASH score*models including 38 patients consecutive referred to a department of occupational medicine, were tested by linear regression analysis.

## Discussion

This study shows that patients with HAVS experience a loss in hand function, measured by the DASH questionnaire. The mean DASH score was 41.2; much higher than what is considered normative values in a US adult population, where the mean score has been reported to be 10 [25]. We found an association between the DASH score, reduced hand-grip strength and tendinitis. The individual answers to the DASH questionnaire showed that several tasks related to everyday life were affected in the HAVS patients. This is valuable information as it indicates that HAVS influences not only the work ability of the patients, but also their leisure time. The patients can stop working, but they still have to cope with tasks at home requiring a certain functional ability in hand/arm.

To our knowledge, there are no previous studies describing the individual DASH scores. However, there are studies describing the effect of hand-arm vibration on the ability to perform daily activities. A Swedish study of 105 male workers [21] found that 42% experienced difficulty performing at least one daily activity as measured by the Evaluation of Daily Activities Questionnaire (EDAQ). The most difficult items apart from tasks involving work-related activities were heavy gardening and opening a jar with a screw lid. This is comparable to our study where 86% of the patients were affected in these two areas, and 39% and 40% respectively, experienced severe difficulty or inability to perform these activities.

The results of the Stockholm workshop staging confirms that these patients are severely affected. 34% were classified as stage 3 both in the vascular and the sensorineural scale.

There are some limitations to the study. The number of patients was unfortunately relatively low, reducing the statistical power. This might explain the lack of association between the DASH score and age, smoking, years of vibration exposure, the SWS scales and manual dexterity as measured with the Grooved Pegboard. The lack of association between the DASH score and age is in contrast to other HAVS studies [16,18,19]. For the other variables the picture is more diverse, and our study can confirm the lack of association to the Stockholm vascular stage found in other studies [16-18].

The cause of the tendinitis is not explored in the present study. This diagnosis might be caused by exposure to vibration, but could also be caused by a number of other factors. However, what is interesting, is that the diagnosis is present in a high number of these HAVS patients, and is likely to play a role for the disability.

All patients diagnosed with HAVS at our department in a five-year period were invited, and a high proportion (72%) agreed to participate. The high response rate strengthens the study. The study did not include any control group, but our intent was to describe disability and study associations between disability and different measures of exposure, symptoms and clinical outcomes in a group of HAVS patients, not to make comparisons. Another strength of the study was that all patients were examined by the same team at our outpatient clinic, minimizing any interpersonal variation in methods.

The DASH questionnaire was – for some of the patients– completed several years after the clinical examination. This would be a major drawback if our intent was to study acute health effects of vibration. In this study the subjects had a definite diagnosis of HAVS at the time of clinical examination and we judge that the delay in time before completion of the DASH questionnaire is less important. Also, the majority of the patients were assessed 2 years or less prior to filling in the questionnaire. When the questionnaire was completed, 50% of the patients were out of work due to health problems, and consequently were no longer exposed to ergonomic stressors including vibration. One would expect a certain degree of improvement after work cessation [8,26], but as the DASH score was higher among those falling out of work, this confirms that HAVS patients experience a lasting disability independent of current exposure. There could be additional factors influencing the patients’ coping strategies; mental conditions might play a role. Depression and anxiety in combination with chronic musculoskeletal pain enhances the disability in the patients [27]. A Swedish study found age and psychological mood to be the strongest predictors of work ability in patients with suspected HAVS [28]. The possible co-existence of HAVS, anxiety and depression has not been focused in our study.

The major findings in this study are in agreement with previous studies. The DASH score in our study population is similar to findings in other studies of HAVS patients [15,18].

A Swedish study compared patients with traumatic hand injury with HAVS patients [15]. The DASH score for these two groups were 22 and 38 respectively, i.e. slightly lower than in our study. The HAVS patients in the Swedish study had a median vibration exposure of 29 years, somewhat longer than the patients in our study who had an average exposure of 22.4 years. This might be due to different exposure levels. The authors describe that the severity of symptoms and influence on daily life was most apparent in patients with HAVS.

In a Canadian study published in 2009 the average DASH score was 42.2 and the authors state that “workers with HAVS have significant upper extremity disability” [18]. This study also found an association between DASH score and several variables, among which upper extremity pain score had the strongest effect. Since upper extremity pain is considered the most common musculoskeletal symptom caused by hand-arm vibration [3], the authors conclude that musculoskeletal factors are the largest contributors to the disability found in their study. This is in agreement with our findings as tendinitis was the variable with greatest impact on the DASH score.

Whether this association is due to vibration exposure per se or is caused by other ergonomic factors is not clarified. Workers exposed to hand-arm vibration are also exposed to demanding work situations including lifting of heavy weights, working in bent and twisted postures, and work demanding static muscle contraction [14,29]. This often makes it hard to separate the impact of vibration from the impact of other ergonomic stressors on the human body.

The hand-arm problems also affected the answers to a question reflecting the patients self-esteem, as 63% agreed (or strongly agreed) with the statement measuring this quality.

Few studies address this issue, but a British study published in 2010 describes interviews with nine HAVS patients, all men [30]. They conclude – in agreement with our study – that people diagnosed with HAVS often experience impacts on individual self-worth.

## Conclusion

Our study shows that patients with HAVS experience difficulties in everyday life concerning both physical and psychological health. The patients experienced extensive and severe symptoms. This illustrates the need for increased focus on the condition to prevent new cases and to detect the disease at earlier stages. The legislation in this area in Norway is similar to that in the EU countries and unequivocal when it comes to protecting workers from the adverse effects of mechanical vibration. The implementation is, however, lacking at present, and requires more attention from the authorities.

Further studies on the subject would contribute to an increasing awareness of this condition both in Norway and in other countries. Larger studies are suggested. As there are relatively few patients referred at the regional hospitals, multi-centre studies might be advised.

## Abbreviations

HAVS: Hand-arm vibration syndrome; DASH questionnaire: The disabilities of the arm, shoulder and hand questionnaire; SWS: The Stockholm workshop scales; CTS: Carpal tunnel syndrome; EDAQ: Evaluation of daily activities questionnaire.

## Competing interests

The authors declare that they have no competing interests.

## Authors’ contributions

KB has planned and carried out the study, performed data analysis and written the article. BEM has contributed in data analysis and writing of the article. ÅI has performed data analysis as well as read and commented on the article. All authors read and approved the final manuscript.
